# Decreased Interoceptive Awareness as a Risk Factor for Moderate to Severe Pain in Japanese Full-Time Workers: A Longitudinal Cohort Study

**DOI:** 10.3390/jcm12082896

**Published:** 2023-04-16

**Authors:** Saki Takaoka, Kenta Wakaizumi, Chisato Tanaka, Shintaro Tanaka, Morihiko Kawate, Reiko Hoshino, Ko Matsudaira, Daisuke Fujisawa, Hiroshi Morisaki, Shizuko Kosugi

**Affiliations:** 1 Department of Anesthesiology, Keio University School of Medicine, 35 Shinanomachi, Shinjuku-ku, Tokyo 160-8582, Japan; 2 Interdisciplinary Pain Center, Keio University Hospital, Tokyo 160-0016, Japan; 3 Department of Neuropsychiatry, Keio University School of Medicine, 35 Shinanomachi, Shinjuku-ku, Tokyo 160-8582, Japan; 4 Department of Pain Medicine, Fukushima Medical University School of Medicine, Fukushima 960-1295, Japan

**Keywords:** interoceptive awareness, multidimensional assessment of interoceptive awareness (MAIA), chronic pain, emotional stability, exercise

## Abstract

Interoceptive awareness, the conscious perception of internal bodily states, is a key construct of mind-body interaction. Decreases in interoceptive awareness, as measured by the Multidimensional Assessment of Interoceptive Awareness (MAIA), are found in chronic pain patients. In this study, we explored whether a specific aspect of interoceptive awareness is a risk for the onset and chronicity of pain. A longitudinal cohort study was conducted in 2018 and 2020 among a sample of full-time workers in an industrial manufacturing company in Japan. Participants completed a questionnaire on pain intensity, MAIA, exercise habits, kinesiophobia, psychological distress and work stress. Principal component analyses using the MAIA identified two principal components: *self-control* and *emotional stability*. Low *emotional stability* was associated with the prevalence of moderate to severe pain in 2020 among people with mild or no pain in 2018 (*p* < 0.01). Lack of exercise habits were associated with the prevalence of moderate to severe pain in 2020 among people with pain in 2018 (*p* < 0.01). Furthermore, exercise habits were associated with reduction in kinesiophobia among people with moderate to severe pain in 2018 (*p* = 0.047). Overall, these findings indicate that low *emotional stability* may be a risk for the onset of moderate to severe pain; lack of exercise habits may sustain kinesiophobia and be a risk for the chronicity of pain.

## 1. Introduction

Chronic pain is a common heath problem in modern society. In a Japanese national survey, lower back pain, stiff shoulders, joint pain, and headaches were reported to be among the most common subjective symptoms [1]. Although these pain symptoms may not be lethal conditions, they negatively impact one’s activities of daily living and trigger various psychological problems, such as fear of pain, anxiety, and depression [2,3,4]. Together with these physical and psychological problems, the negative impact of chronic pain extends into the work performance of an individual [5,6]. We reported that pain severity itself is an independent risk factor for reduced performance (presenteeism) and absence from work (absenteeism) in a sample of Japanese full-time workers [7]. Investigations of chronic pain, especially in the working population, is an issue that requires urgent attention to sustain stable work productivity.

While the underlying mechanisms remain widely unresolved, advances in pain research have revealed various biological, psychological, and socio-demographic variables that lead to the onset of chronic pain [8,9,10,11]. However, since these variables dynamically relate to each other and cannot be distinctly separated, the impact of interaction between bodily and psychological states (mind-body interaction) should not be disregarded.

A key construct of the mind-body interaction is known as interoceptive awareness. Interoceptive awareness is the conscious perception of our internal state and bodily sensations (interoception), such as pain, heartbeat, and respiration, that create our sense of physical self. It is a multifaced construct reflecting one’s sensitivity, emotional response, and attention styles towards his or her bodily experiences [12]. The Multidimensional Assessment of Interoceptive Awareness (MAIA) is a self-reported questionnaire that has been developed to capture the complexity of an individual’s interoceptive awareness from multiple dimensions [13]. A randomized control trial identified that mindfulness exercises, including Yoga, increase the MAIA scores, suggesting that the MAIA scores reflect interoceptive awareness [14].

Growing evidence implies that alterations in interoceptive awareness, as measured by MAIA, are found in patients with chronic pain [15]. The cross-sectional study also identified the MAIA scales that are associated with psychological and pain-related variables, including perceived stress, depression, fear of harm, and catastrophizing. However, to date, the relationship between the MAIA and the onset and chronicity of pain has not been investigated. In this study, we report longitudinal data on the association of interoceptive awareness and pain in a sample of Japanese full-time workers. The specific aim of the study was to explore whether a specific aspect of interoceptive awareness, as measured by the MAIA, may be a risk for the onset and chronicity of pain.

## 2. Methods

A two-wave longitudinal study was conducted in 2018 and 2020 in a branch office of an industrial manufacturing company in Japan. The office was a technology development division of the company, where most employees were desk workers. In the first survey, a set of self-reported questionnaires was distributed to all full-time employees by the company’s health care administration team in July 2018. The details of the survey are described elsewhere [7]. In the second survey, a similar set of questionnaires was sent by post in December 2020 to 349 employees who completed the first survey. In both surveys, participants were asked to fill out the questionnaires and to return the completed form to our research unit by post within a month.

### 2.1. Participants

We included workers (1) with full-time employment, (2) who consented to provide their annual health check data, and (3) who were able to understand and complete the questionnaires in Japanese. We only included full-time workers because part-time employees vary in their working hours and were therefore considered unsuitable as research subjects. All subjects were informed about the aim of the study through the company’s intranet and a document attached to the questionnaire. Subjects were informed that participation in the survey was voluntary, and that submission of the completed questionnaire would be taken as an informed consent to participate.

### 2.2. Measurements

The self-reported questionnaire consisted of the following items:

#### 2.2.1. Pain Intensity

Participants reported the presence and intensity of pain within four weeks preceding the survey using the 0–10 Numerical Rating Scale (NRS), where ‘0’ indicated no pain and ‘10’ indicated the worst pain imaginable [16]. NRS scores of ≤2 were defined as “slight or no pain”, and NRS scores of ≥3 were defined as “moderate to severe pain” in this study, corresponding to a median of the pain intensity.

#### 2.2.2. Interoceptive Awareness

Participants’ levels of interoceptive awareness were assessed with the Japanese version of the Multidimensional Assessment of Interoceptive Awareness (MAIA) [13,17]. Of the eight domains of MAIA, we selected five domains (*attention regulation*, *self-regulation*, *trusting*, *not distracting*, and *not worrying*) that were previously shown to be associated with pain-related variables, such as stress and depression, in a study by Mehling that assessed the correlation between MAIA scales and psychological and pain-related variables in people with post or current low back pain. The other three domains (*body listening*, *emotional awareness*, and *noticing*) were not related to any of the psychological or pain-related measures [15]. Each domain consisted of three to four items, which were rated on a six-point scale of frequency from 0 (never) to 5 (always), and scores were averaged. Internal consistency for each domain has been reported as follows; Cronbach’s α = 0.66 (*not distracting*), 0.67 (*not worrying*), 0.87 (*attention regulation*), 0.83 (*self-regulation*), and 0.79 (*trusting*) [13]. A higher score indicated a greater confidence and awareness in one’s bodily experiences.

#### 2.2.3. Psychological Distress

Participants’ psychological distress levels were assessed via the Japanese version of the Kessler Psychological Distress Scale (K6), a well-validated questionnaire (Cronbach’s α = 0.85) [18,19,20]. Participants were asked to rate their psychological distress during the past 30 days on six items of K6 (e.g., nervousness and worthlessness) on a five-point scale of frequency from 0 (never) to 4 (always). A higher total score indicated more intense psychological distress. In accordance with previous studies, we categorized participants who scored 13 points or more as having serious psychological distress [20,21].

#### 2.2.4. Fear of Movement

We used the Japanese short version of the Tampa Scale of Kinesiophobia (TSK-J11) to assess pain-related fear of movement [22,23]. TSK is reported to have a strong correlation with the Pain Catastrophizing Scale (PCS), which is a measure that assesses the extent of an individual’s catastrophic thinking [22]. TSK consists of 11 items, each of which are rated on a four-point Likert scale ranging from 1 (strongly disagree) to 4 (strongly agree) with sufficient internal consistency (Cronbach’s α = 0.92). The total score was obtained by summing the scores for the 11 items. A higher total score indicated a greater fear of movement.

#### 2.2.5. Work Stress

The participants’ work-related stress was evaluated based on the job demand-resource model, which indicates that high work demand and limited work resources lead to work-related mental strain [24]. Work demand is expressed by work overload and work-related emotional demands. We evaluated the participants’ work overload using four items (Cronbach’s α = 0.88) that refer to a demanding workload (e.g., job quantity) and high pressure (e.g., time pressure) [25]. Participants’ work-related emotional demands were evaluated using six items (Cronbach’s α = 0.86) developed by van Veldhoven that refer to the frequency of emotionally challenging events in one’s job circumstances [26]. All items were rated on a five-point scale of frequency from 1 (never) to 5 (always), where a higher total score represented a higher work demand.

To evaluate the participants’ available resources at work, we used the task controllability subscale of the Brief Job Stress Questionnaire (BJSQ) [27]. The BJSQ is an instrument developed by the Japanese Ministry of Health, Labor and Welfare and its research group, and it has been widely used in Japan as a regular yearly screening for high psychological stress in the workplace. In this questionnaire, personal controllability at work was assessed by three items on a four-point scale of frequency that ranged from 1 (always) to 4 (never). A higher total score represented lower controllability at work.

The total score of work overload, work-related emotional demand, and controllability was defined as work stress in this study.

#### 2.2.6. Home Stress

From the viewpoint of a work-home interference, we investigated the participants’ home stress. We assessed participants’ home demands on an eight-item questionnaire with a five-point scale referring to overload and emotional demands at home. A higher total score indicated a greater home demand [28]. Cronbach’s α of the home overload and home emotional demands were 0.80 and 0.76, respectively. Controllability at home was assessed in accordance with the evaluation of work controllability, using four items on a five-point scale ranging from 1 (always) to 5 (never). A higher total score indicated lower controllability. The total score of home demand and controllability was defined as home stress in this study.

#### 2.2.7. Demographic and Lifestyle Related Measures

Participants’ sociodemographic and health-related characteristics were collected from their latest annual health check data. The following data were collected: age, sex, body mass index (BMI, kg/m^2^), highest educational level achieved (high school graduate, junior college graduate, bachelor’s degree, master’s degree, or doctoral degree), sleep duration (hours: <5, 5, 6, 7, 8, or ≥9), and exercise habits (whether a participant exercises more than 30 min, twice per week). Low education was defined as bachelor’s degree or lower in this study. We defined short sleep as less than 6 h of sleep per day, because 7–9 h of sleep is considered adequate for a healthy adult [29].

### 2.3. Statistical Analysis

Principal component analysis with an orthogonal rotation was applied to the five domains of MAIA to reduce the dimension. The number of principal components was identified by following criteria: eigen value ≥ 1 and explained variance ≥ 10%. Participants were categorized into those with mild or no pain and those with moderate to severe pain based on their pain intensity in 2020. The demographic data in 2018 was compared between the two groups. We performed a multivariable logistic regression analysis, in which the measures in 2018 were used as independent variables, to identify risk factors for the prevalence of moderate to severe pain in 2020. Participants were then stratified into two groups based on their pain intensity in 2018, and risk factors for moderate to severe pain in 2020 were analyzed in each group to differentiate between the risks for new onset pain and risks for chronicity of the moderate to severe pain. In addition, we analyzed association of exercise habits with changes in the psychological factors and five domains of the MAIA in people with moderate to severe pain in 2018 using a repeated measure analysis of variance (rm-ANOVA). All statistical analyses were performed by the JMP^®^ Version 16.0.0 (SAS Institute) software package. Statistical significance was identified by two-tailed *p* values < 0.05.

## 3. Results

**Figure 1:**
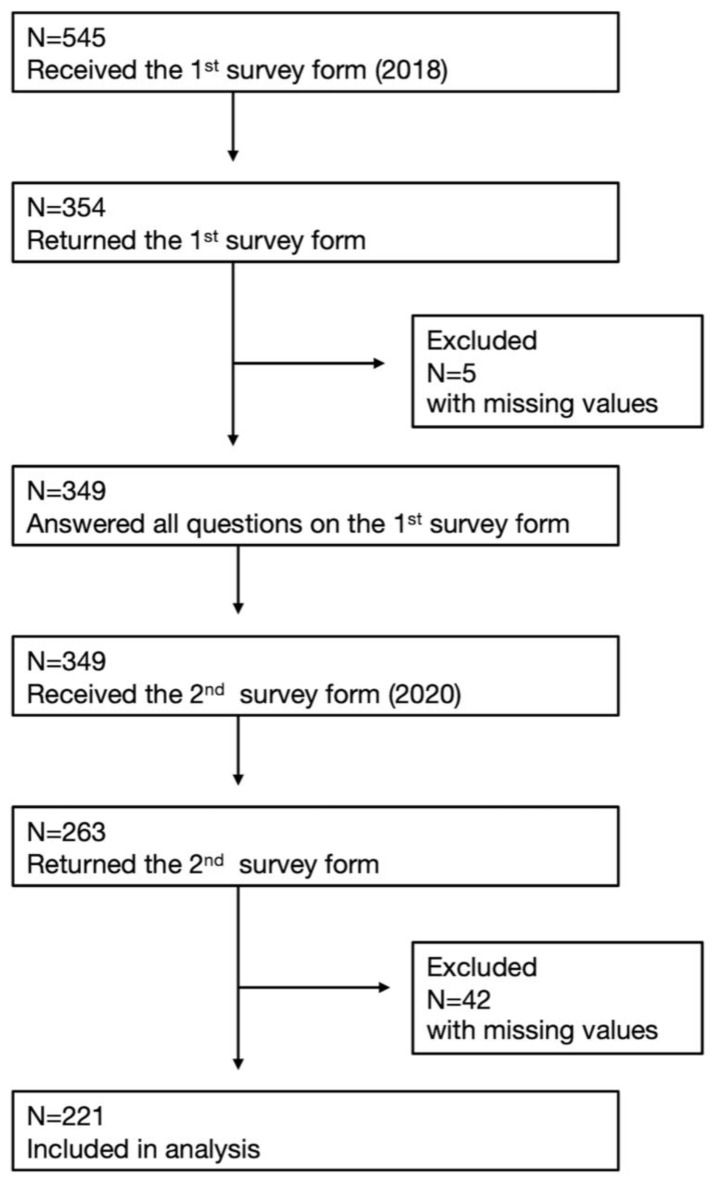
Flow Diagram of Study Participants.

All 545 employees in the office were invited to participate in the study. Of those, 349 employees responded to the first survey with a complete set of data (64.0% response rate). Among those who responded to the first survey, 263 employees responded to the second survey (75.4% response rate). We excluded 42 responses with incomplete data, and a total of 221 participants were included in the analyses (Figure 1). The mean age of the participants was 41.4 years in 2018, ranging from 18 to 62 years, and the number of male participants was 185 (83.7%).

Table 1 shows the characteristics of participants in the 2018 survey, comparing the two groups divided according to their reported pain intensity in 2020. Participants with moderate to severe pain in 2020 (*n* = 114, 51.6%) were more likely to be female (*p* = 0.046) and with lower *not distracting* and *not worrying* scores on the MAIA (*p* = 0.004 and 0.042, respectively) compared to those with mild or no pain (*n* = 107, 48.4%) at the baseline. There were no statistically significant differences between the two groups in age, BMI, work stress, home stress, TSK, or in other domains of the MAIA. Likewise, no significant differences were found in the number of participants with low education, short sleep periods, high psychological distress, and lack of exercise habits.

**Figure 2:**
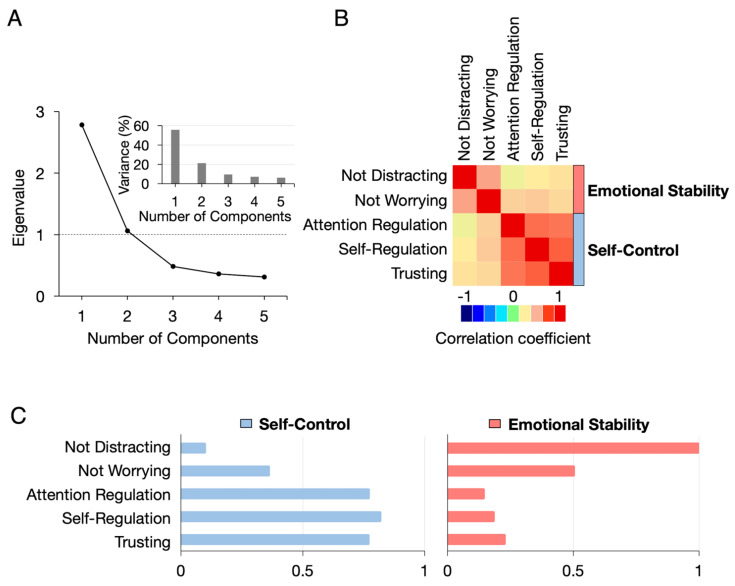
**Principal component analysis of the five MAIA domains.** (**A**) A screen plot of eigenvalues and a bar graph of explained variances. Number of principal components was identified by criteria of 1) eigenvalue ≥ 1 and 2) explained variance ≥ 10. (**B**) Heatmap of correlation coefficients of measures. (**C**) Loadings of measures to the identified two principal components.

In the principal component analysis, we identified two principal components with eigenvalues greater than 1.0. The first principal component was named “*self-control*” corresponding to the most relevant measures, which were *attention regulation*, *self-regulation*, and *trusting,* with more than 0.5 of the absolute loading value. The second component, “*emotional stability*,” was represented by *not distracting* and *not worrying* (Figure 2).

A multivariable logistic regression analysis showed that the prevalence of moderate to severe pain in 2020 was associated with the female sex and lower *emotional stability* in 2018 (*p* = 0.025 and 0.006) (Table 2). In the stratified analyses, the significant association between *emotional stability* and prevalence of moderate to severe pain in 2020 remained only in those who reported mild or no pain in 2018 (*p* < 0.01), suggesting low *emotional stability* may be a risk for new onset pain. On the other hand, lack of exercise habits in 2018 was significantly associated with higher prevalence of moderate to severe pain in 2020 in people with pain in 2018 (*p* < 0.01), suggesting lack of exercise habits may be a risk for the chronicity of pain (Table 3). Rm-ANOVA identified a significant interaction effect between exercise habits and improvements in TSK scores (F = 4.056, *p* = 0.047), indicating a reduced fear of movement due to exercise habits may help recovery and transitioning from moderate or severe pain to slight or no pain (Table 4).

## 4. Discussion

The present study is a longitudinal investigation of chronic pain and interoceptive awareness among a sample of Japanese full-time workers. The results revealed low *emotional stability* of the MAIA as a risk factor for new onset of pain among workers with slight or no pain. In addition, although no significant relationship between MAIA and the chronicity of pain was observed, lack of exercise habit was revealed as a risk factor for chronicity of moderate to severe pain in this population. Furthermore, we found that regular exercise habits may reduce fear of movement and help prevent such chronicity of pain.

### 4.1. Association of Emotional Stability and the Onset of Pain

Decrease in *not distracting* and *not worrying*, the two major domains of *emotional stability*, represent one’s tendency to distract oneself from and worry about sensations of pain, respectively. *Not distracting* has been known to strongly negatively associate with a coping style based on ignoring one’s pain, which is the tendency to suppress pain-related negative thoughts and experiences [15]. Past studies suggest that thought suppression is associated with elevated emotional distress and depression [30]. The *not worrying* scale of MAIA, on the other hand, has been shown to negatively correlate with pain catastrophizing [15]. It is well established that catastrophic cognition, an excessive worry and fear of pain, plays a significant role in the amplification of pain, and has a link to depression [31].

Past studies have postulated that individual differences in pain coping styles have an important impact on pain-related outcomes [11]. Therefore, it may be possible that individuals with low *emotional stability* are prone to exhibit these maladaptive patterns of pain coping when facing the experience of pain and are at a risk of experiencing stronger emotional distress in the state of pain, leading to the onset of heightened pain.

In contrast, nonjudgmental and accepting attitudes towards one’s body experience has been shown to alleviate acute as well as chronic pain and are considered as advantageous pain coping styles [32,33]. While some individuals have a greater innate capacity for such attitudes, it can also be obtained through intentional training, such as mindfulness-based cognitive therapy (MBCT) [34,35]. MBCT is a modified form of cognitive behavioral therapy that incorporates mindfulness strategies, such as meditation and breathing exercises, that encourages participants to observe present bodily and sensory experiences in an accepting and non-reactive manner. MBCT intends to change individuals’ habitual behaviors of restricted focus, excessive emotional suffering and ineffective avoidance when facing pain and other distressing bodily sensations [36]. MBCT has been shown to be effective in the treatment of chronic pain [33]. It has been shown to improve all of the eight MAIA subscales in healthy participants [37]. Recent functional and structural MRI studies have shown that pain relief through mindfulness-based interventions is associated with change in the brain processes that are distinct from placebo effects [38,39]. These results imply that mindfulness-based interventions (such as MBCT) may have the potential to raise individuals’ *emotional stability*, leading to prevent the onset of moderate to severe pain.

### 4.2. Prevention of Pain Chronicity

The efficacy of physical exercise on chronic pain, even for patients with severe pain, has been well documented in numerous studies [40,41] While the underlying mechanisms of exercise-mediated pain relief are not entirely understood, the beneficial effect of exercise on pain does not depend solely on improvements in physical function (e.g., strength, muscular endurance, range of motion) [42], but also depends on other factors. Recent studies indicated that exercise-induced changes in psychological factors, such as pain catastrophizing and self-efficacy, play a mediating role in pain alleviation [43,44]. Moreover, Kernan observed statistically significant improvements in pain and measures of kinesiophobia in patients with chronic low back pain after six weeks of physical therapy programs in which exercises were performed in a quota-based manner [45]. This finding is consistent with those reported in our study, suggesting that regular exercise may reduce fear of movement in people with pain, resulting in pain alleviation through improvement of physical and psychological function.

### 4.3. Study Limitations

The present study has several limitations. First, among the participants who reported having moderate to severe pain both in 2018 and 2020, there is a possibility that we are not observing a continued presence of the same pain but are observing new onsets of pain symptoms at two time points. However, we can still assume that a common central process leading to pain chronicity exists in such cases, indicating that the findings of this study suggest vulnerability to pain symptoms. Secondly, this study was conducted at a single workplace in which most workers were predominantly male and desk-workers, so the results cannot be generalized to the general population. Further study is required to confirm these findings in other settings and types of populations. Thirdly, due to the small sample size, we could not stratify participants by the location of pain in this study. However, as shown in our previous study using same data obtained in 2018 [7], many participants had complained of frequent locations of pain symptoms, i.e., neck and shoulder pain, low back pain, knee pain, and headaches. Therefore, our findings are possibly acceptable in a general population. Finally, *emotional stability* is a novel domain of the MAIA used in this study. While it seems to represent the psychological vulnerability in individuals in situations of pain or discomfort, verification of its validity will be our next topic of concern.

## 5. Conclusions

For participants with slight or no pain, sustained *emotional stability* may be a resistant force to developing moderate to severe pain. For people with moderate to severe pain, lack of exercise habits may sustain fear of movement and inhibit recovery from pain. This study suggests stratified approaches are more likely to be effective for each group of participants with and without moderate to severe pain. Further longitudinal interventional study is warranted on the question of whether health promotion efforts in the workplace, such as mind-body intervention and physical activity intervention can act as prevention for the onset of chronicity of pain.

## Figures and Tables

**Table 1:** Group comparisons of demographic characteristics in 2018 between the categories of pain intensity in 2020.

Pain Intensity in 2020	Slight or No Pain		Moderate to Severe Pain		*t* or *Z*	*p*	
**N**	107		114				
**Age (years), mean (SEM)**	40.87	(1.07)	41.8	(1.03)	0.64	0.521	
**Male, n (%)**	95	(88.79)	90	(78.95)	3.99	0.046	*
**BMI (kg/m^2^), mean (SEM)**	23.12	(0.31)	23.1	(0.30)	0.00	0.999	
**Low education, n (%)**	28	(26.17)	27	(23.68)	2.34	0.673	
**Short sleep (< 6 h/day), n (%)**	73	(68.22)	78	(68.42)	0.00	0.975	
**Work stress, mean (SEM)**	30.59	(0.82)	32.3	(0.79)	1.53	0.128	
**Home stress, mean (SEM)**	22.54	(0.46)	23.2	(0.45)	1.06	0.292	
**TSK, mean (SEM)**	22.92	(0.46)	23.3	(0.45)	0.57	0.572	
**Psychological distress (K6 ≥ 13), n (%)**	11	(10.28)	11	(9.65)	0.03	0.876	
**MAIA, mean (SEM)**							
**Not distracting**	3.25	(0.12)	2.8	(0.12)	−2.90	0.004	**
**Not worrying**	3.00	(0.09)	2.7	(0.09)	−2.05	0.042	*
**Attention regulation**	2.99	(0.09)	2.8	(0.09)	−1.14	0.255	
**Self-regulation**	2.92	(0.10)	2.8	(0.09)	−1.14	0.254	
**Trusting**	2.92	(0.10)	2.8	(0.10)	−1.12	0.265	
**No exercise habit, n (%)**	28	(26.17)	43	(32.13)	3.40	0.065	

Moderate to severe pain was defined as three or more in the numerical rating scale of averaged pain intensity during the latest four weeks in the second survey. Group comparisons were performed using unpaired *t*-test for continuous variables or chi-square tests for categorical ones. SEM: standard error of the mean, BMI: body mass index, TSK: Tampa scale for kinesiophobia, K6: Kessler psychological distress scale, MAIA: multidimensional assessment of interoceptive awareness. * *p* < 0.05, ** *p* < 0.01.

**Table 2:** Risk factors associated with prevalence of the moderate to severe pain in 2020 (N = 221).

	Odds Ratio	95% CI (LL, UL)	*p*	
**Age**	1.028	(0.999, 1.057)	0.053	
**Male**	0.368	(0.154, 0.881)	0.025	*
**Work stress**	1.025	(0.988, 1.064)	0.190	
**Home stress**	0.992	(0.933, 1.055)	0.804	
**TSK**	0.984	(0.923, 1.049)	0.620	
**Psychological distress**	0.453	(0.163, 1.256)	0.128	
**Emotional stability**	0.640	(0.463, 0.887)	0.006	**
**Self-control**	0.853	(0.612, 1.188)	0.345	
**No exercise habit**	1.719	(0.924, 3.198)	0.087	

Multivariable logistic regression analysis was performed. CI: confidence intervals, LL: lower limit, UL: upper limit, TSK: Tampa scale for kinesiophobia. * *p* < 0.05, ** *p* < 0.01.

**Table 3:** Stratified analyses based on pain intensity in 2018 for risk factors associated with prevalence of the moderate to severe pain in 2020.

	Slight or No Pain in 2018 (N = 111)				Moderate to Severe Pain in 2018 (N = 110)			
	Odds Ratio	95% CI (LL, UL)	*p*		Odds Ratio	95% CI (LL, UL)	*p*	
**Age**	1.052	(1.003, 1.102)	0.030	*	1.016	(0.974, 1.059)	0.467	
**Male**	0.346	(0.066, 1.809)	0.209		0.441	(0.129, 1.511)	0.193	
**Work stress**	1.033	(0.980, 1.088)	0.229		1.039	(0.973, 1.109)	0.242	
**Home stress**	0.917	(0.829, 1.015)	0.094		1.035	(0.944, 1.135)	0.464	
**TSK**	0.917	(0.820, 1.025)	0.123		0.966	(0.879, 1.062)	0.475	
**Psychological distress**	0.449	(0.108, 1.858)	0.269		0.657	(0.098, 4.410)	0.665	
**Emotional stability**	0.406	(0.236, 0.699)	0.001	***	1.046	(0.606, 1.803)	0.872	
**Self-control**	0.790	(0.473, 1.319)	0.365		1.094	(0.633, 1.891)	0.747	
**No exercise habit**	0.974	(0.359, 2.643)	0.959		3.956	(1.391, 11.255)	0.010	**

Multivariable logistic regression analysis was performed. CI: confidence intervals, LL: lower limit, UL: upper limit, TSK: Tampa scale for kinesiophobia. * *p* < 0.05, ** *p* < 0.01, *** *p* < 0.001.

**Table 4:** Associations of exercise habit with changes in psychological factors in people with moderate to severe pain in 2018 (N = 110).

	2018		2020		No Exercise Habit			Time			No Exerice Habit × Time		
	Mean	(SEM)	Mean	(SEM)	F	*p*		F	*p*		F	*p*	
**TSK**	23.99	(0.48)	22.85	(0.58)	7.715	0.007	**	4.737	0.032	*	5.131	0.026	*
**Work stress**	31.89	(0.80)	31.12	(0.82)	0.199	0.656		0.138	0.711		0.220	0.640	
**Home stress**	23.35	(0.49)	23.80	(0.47)	0.030	0.863		0.549	0.460		0.001	0.980	
**MAIA**													
**Attention regulation**	2.88	(0.09)	2.89	(0.10)	0.015	0.904		0.076	0.784		0.038	0.846	
**Self-regulation**	2.81	(0.09)	2.82	(0.10)	3.735	0.056		0.025	0.876		0.014	0.906	
**Trusting**	2.75	(0.10)	2.77	(0.10)	4.303	0.041	*	0.233	0.630		0.155	0.694	
**Not distracting**	2.84	(0.11)	2.98	(0.12)	0.850	0.359		0.160	0.690		1.688	0.197	
**Not worrying**	2.78	(0.09)	2.68	(0.09)	0.751	0.388		0.277	0.600		0.278	0.599	

Repeated measure analysis of valiance was performed, adjusted for age, sex, body mass index, and psychological distress. SEM: standard error of the mean, TSK: Tampa scale for kinesiophobia, MAIA: multidimensional assessment of interoceptive awareness. * *p* < 0.05, ** *p* < 0.01.

## Data Availability

Data are available upon reasonable request. Analyzed data in this study are available under the permission of the Institutional Review Board in Keio University School of Medicine corresponding to each request (https://www.ctr.med.keio.ac.jp/rinri/ (15 April 2023)).
